# Comparing Wound Healing and Infection Risk Between Early and Late Dressing Removal After Abdominal Hysterectomy

**DOI:** 10.7759/cureus.62535

**Published:** 2024-06-17

**Authors:** Emrullah Akay, Kübra Irmak, Ravza Incebıyık, Fatma Sağlam, Enes Burak Mutlu

**Affiliations:** 1 Obstetrics and Gynecology, Başakşehir Çam and Sakura City Hospital, Istanbul, TUR; 2 Obstetrics and Gynecology, Istinye University, Istanbul, TUR

**Keywords:** postoperative complications, ­wound healing, dressing timing, hysterectomy, eras protocols

## Abstract

Introduction: This study evaluates the effects of dressing timing after abdominal hysterectomy on wound healing and infection risk. It highlights the potential for early dressing removal to accelerate healing and underscores the need for clear guidelines in wound care that align with the ERAS (Enhanced Recovery After Surgery) protocol.

Methods: Using a prospective, randomized, double-blind design, this research was carried out at Başakşehir Çam and Sakura City Hospital, Istanbul, Turkey. The objective was to investigate the impact of early dressing removal on wound healing and infection rates after elective abdominal hysterectomy.

Results: Demographic parameters such as age, height, weight, and body mass index (BMI) were found to have no significant impact on wound healing. Patients whose dressings were removed early had shorter hospital stays. No significant differences were observed between the two groups in terms of wound complications and hospital readmission rates.

Conclusions: Early dressing removal after abdominal hysterectomy was observed to positively affect wound healing and facilitate earlier hospital discharge. However, no significant differences were found in hospital readmission rates between the two groups. These findings suggest that the dressing timing can be more flexible within the ERAS protocol and does not have a decisive impact on postoperative complications.

## Introduction

The wound-healing process is a complex biological sequence initiated by the body after injury, encompassing stages of inflammation, proliferation, and maturation. The inflammatory phase begins immediately after injury, as the body sends blood cells to the wound site to combat infection and provide the cells and nutrients necessary for healing. During the proliferation phase, new tissue forms, and the edges of the wound merge, while the maturation phase strengthens the new tissue and closes the wound. Typically, this process lasts four to six weeks; however, chronic wounds may not heal within this time frame and can be compromised by factors such as hypoxia, bacterial colonization, and ischemia [1,2].

In the management of surgical wounds, the timing and type of dressing can influence the healing process and the risk of infection. Early removal of dressings can improve patient comfort and support early discharge [3]. However, the findings in the literature on the impact of dressing removal timing on surgical site infections (SSI) are contradictory [4,5].

Hysterectomy is a common gynecological procedure, often performed for conditions such as benign uterine fibroids. Treatment options should be personalized according to the individual's condition [6]. Abdominal hysterectomy, in particular, may be preferred in the presence of large fibroids or expanded diseases in pelvic structures, providing a broad surgical field [7].

The ERAS (Enhanced Recovery After Surgery) protocol is a multidisciplinary approach designed to accelerate postoperative recovery and facilitate the return of patients to normal life more quickly. However, within the ERAS protocol, there are no clear recommendations regarding the timing of postoperative wound dressing removal and dressing selection [8].

This study investigates the optimal timing of dressing removal after abdominal hysterectomy and the risk of infectious complications, focusing particularly on the effects of early (12 hours) versus late (48 hours) dressing removal on clinical outcomes. Given the gap in the current literature, this research could provide significant contributions and valuable insights for optimizing post-surgical wound care practices.

The study aims to thoroughly analyze the effects of dressing timing on wound healing and infection risk after abdominal hysterectomy, determining the potential benefits and risks of early dressing removal. The research assesses whether early removal accelerates wound healing, reduces infection risk, and affects the overall recovery process of the patient. In addition, it provides data on how early dressing removal influences hospital stay duration and healthcare costs. This study could offer crucial information to improve postoperative wound care practices.

## Materials and methods

Study design and execution

This prospective, randomized, controlled study was conducted at Istanbul City Hospital between November 2021 and April 2024. Two hundred women over the age of 30 years who underwent elective abdominal hysterectomy due to treatment-resistant abnormal uterine bleeding, myomas, and adnexal masses were included in the study. Participants gave their written consent after receiving detailed information about the study objectives, procedures, and potential risks.

Research question and hypotheses

The study aimed to investigate the impact of early dressing removal following elective abdominal hysterectomy on wound healing and infection rates. The hypothesis posited that early dressing removal accelerates wound healing and reduces infection rates.

Randomization process

Randomization was a critical step to ensure equal chances of assignment to the experimental or control group. This process was executed using Random Allocation Software 2 (Informer Technologies, Inc., Altamor Drive, LA), which employs an advanced algorithm to randomly and unbiasedly distribute participants into groups, considering their demographic and clinical characteristics. Each participant was assigned a unique identifier number, ensuring a double-blind setup to maintain the impartiality of the randomization process.

Data collection

Data were collected from hospital records and face-to-face interviews. Furthermore, demographic and clinical information of participants was collected using standardized forms. The data collection process was conducted ethically, safeguarding patient confidentiality.

Exclusion criteria

Preoperative exclusions included people with known immune deficiencies, diabetes diagnoses, recent antibiotic therapy within the last 30 days, and allergies to specific antibiotics. Intraoperative exclusions included patients with chronic tuboovarian abscesses, those with dense abdominal adhesions, and those who developed bowel or urinary complications. Postoperative exclusions were patients admitted to intensive care, those who required more than four units of red blood cell transfusion due to postoperative bleeding, and patients undergoing relaparotomy.

Demographic and clinical information of the participants

Demographic data from participants, including age, height, weight, Body mass index (BMI), number of births, abortions, and curettages, smoking status, presence of additional diseases, indications for hysterectomy, history of surgeries, preoperative and postoperative hemoglobin and hematocrit levels, and duration of hospital stay, were collected using standard forms.

Study population

In the beginning, consent was obtained from 252 patients. However, after wound evaluation, 52 patients were excluded from the study due to hematoma, wound dehiscence, surgical site infection, and seroma, as well as the inability to perform second follow-up evaluations. The remaining 200 patients were divided into two groups: 100 had their dressing removed 12 hours after surgery and 100 had theirs removed 48 hours after surgery. The selection of patients was based on predefined exclusion criteria.

Initial Evaluation and Complication Categorization

The initial dressing evaluation was performed by resident doctors and specialist physicians in the Department of Obstetrics and Gynecology. Women with complications such as infection or wound disruption were excluded from the study. According to the hospital’s postoperative home care protocol, all patients received second-generation oral cephalosporin therapy.

Second Evaluation and Complication Categorization

A follow-up evaluation on the 10th postoperative day recorded complications at the wound site. The data were matched using Microsoft Excel (Microsoft® Corp., Redmond, WA).

Third Evaluation and Complication Categorization

The patients were contacted by telephone to record any subsequent hospital visits. These data were also matched using Microsoft Excel.

Data analysis

The data analysis process began with the preparation of the dataset. Missing data were imputed using multiple imputation methods based on the responses of the participants. Outliers were identified using Z scores and excluded from the analysis. All data were processed with Statistical Product and Service Solutions (SPSS, 27.0; IBM SPSS Statistics for Windows, Armonk, NY) and Microsoft Excel.

Operational definitions and criteria

The study defined 'treatment-resistant abnormal uterine bleeding' as chronic uterine bleeding that does not respond to standard treatment methods and negatively affects the patient’s quality of life. BMI is calculated by dividing an individual’s weight by the square of their height and is used as an indicator of general health status.

Ethical approval

The study was reviewed and approved by the Ethics Committee of Başakşehir Çam and Sakura City Hospital on 21 November 2023. The approval number E-96317027-514.10-229612382 was recorded under the file number KAEK/27.09.2023.432. The committee evaluated the study protocol, participant selection, data collection and analysis methods, privacy protection measures, and the overall ethical compliance of the study.

Limitations and strengths of the study

The potential limitations of the study include the reliance on the software algorithm for randomization and the specific criteria for participant selection. The strengths of the study lie in its randomized and controlled design, double-blind structure, and comprehensive data analysis methods. These features improve the reliability and applicability of the study findings.

Statistics

For descriptive statistics of the data, mean, standard deviation, median, minimum, and maximum values, frequencies, and proportions were used. The normal distribution of the variables was assessed using the Kolmogorov-Smirnov and Shapiro-Wilk tests. Independent sample t-tests and Mann-Whitney U tests were used for the analysis of quantitative independent variables, while the chi-square test was preferred for qualitative independent variables. All analyses were performed using SPSS 27.0 statistical software.

## Results

The population studied in our research exhibited a wide distribution in terms of demographic and anthropometric measures such as age, height, weight, and BMI. The age range ranged from 35 to 79 years, with a median age of 48 years, indicating a wide age spectrum within the population. The parity status showed that the majority (90.5%) had previously given birth. The rates of abortion and curettage suggested that most of the patients had not undergone these procedures. Data on smoking habits and the presence of additional illnesses indicated that a large portion of the population did not smoke and did not have additional illnesses (Table 1).

**Table 1:** Demographic and Clinical Characteristics of the Patients The Min and Max represent the range of measurements such as age, height, weight, and BMI; Median and Mean ± SD indicate the central tendency and distribution of these measurements. The “+” and “-” signs denote the presence or absence of specific clinical conditions. Data such as preoperative and postoperative hemoglobin, hematocrit, and hospital stay duration are also presented. This information plays a key role in understanding the scope of the research and the profiles of the participants

Variable	Min	Max	Median	Mean	SD	n	%
Age (years)	35.0	79.0	48.0	47.8	± 6.0	-	-
Height (cm)	130.0	180.0	160.0	160.6	± 6.5	-	-
Weight (kg)	47.0	130.0	75.0	76.3	± 14.5	-	-
BMI (kg/m²)	18.8	50.8	29.3	29.7	± 5.8	-	-
Parities (-)	-	-	-	-	-	19	9.5%
Parities (+)	-	-	-	-	-	181	90.5%
Number of parities	1.0	10.0	2.0	2.6	± 1.4	-	-
Abortion (-)	-	-	-	-	-	156	78.0%
Abortion (+)	-	-	-	-	-	44	22.0%
Number of abortions	1.0	7.0	1.0	1.7	± 1.3	-	-
Curettage (-)	-	-	-	-	-	184	92.0%
Curettage (+)	-	-	-	-	-	16	8.0%
Number of curettages	1.0	3.0	1.0	1.4	± 0.7	-	-
Smoking (-)	-	-	-	-	-	187	93.5%
Smoking (+)	-	-	-	-	-	13	6.5%
Additional disease (-)	-	-	-	-	-	125	62.5%
Additional disease (+)	-	-	-	-	-	75	37.5%
Indications for hysterectomy - Myoma Uteri	-	-	-	-	-	163	81.5%
Indications for hysterectomy - Abnormal Uterine Bleeding	-	-	-	-	-	32	16.0%
Indications for hysterectomy - Adnexal Mass	-	-	-	-	-	5	2.5%
Previous operation (-)	-	-	-	-	-	80	40.0%
Previous operation (+)	-	-	-	-	-	120	60.0%
HGB	-	-	-	-	-	-	-
Preoperative	5.7	15.5	11.6	11.7	± 1.7	-	-
Postoperative	6.5	15.8	10.7	10.8	± 1.5	-	-
HCT	-	-	-	-	-	-	-
Preoperative	21.5	48.8	35.6	35.5	± 4.2	-	-
Postoperative	21.0	45.4	32.3	32.5	± 3.7	-	-
Hospital stay (hour)	45.0	100.0	53.5	53.9	± 10.3	-	-

The most common indications for hysterectomy were myoma uteri (81.5%), treatment-resistant abnormal urine bleeding (16.0%), and adnexal masses (2.5%). The preoperative and postoperative hemoglobin (HGB) and hematocrit (HCT) values provided information on blood levels before and after surgery. The average duration of the operations was observed to be 106.9 minutes (Table 1).

Data presented in Tables 1-2 revealed low rates of postoperative complications. The appearance of hematoma (4%), wound separation (9.5%), superficial SSI (10%), and seroma (11%) were observed. The rate of readmission to the hospital was measured at 7.5% (Table 2).

**Table 2:** Postoperative Complications and Hospital Readmission Rates The table presents a summary of post-surgical complications and rates of readmission to the hospital. For each condition, the number and percentage of negative (-) and positive (+) cases are provided.

Variable	n	%
Hematoma (-)	192	96.0%
Hematoma (+)	8	4.0%
Wound separation (-)	181	90.5%
Wound separation (+)	19	9.5%
Superficial SSI (-)	180	90.0%
Superficial SSI (+)	20	10.0%
Seroma (-)	178	89.0%
Seroma (+)	22	11.0%
Readmission to hospital (-)	185	92.5%
Readmission to hospital (+)	15	7.5%

In our study, comparisons between groups with dressing removal times of 12 hours and 48 hours did not show statistically significant differences in the ages (p>0.05; Table 3). Similarly, no significant differences were observed between the two groups in terms of height and weight parameters (p>0.05; Table 3). Comparison of BMI values also revealed no significant differences between the groups (p>0.05; Table 3).

**Table 3:** Comparison of Demographic and Clinical Outcomes by Dressing Removal Time Following Abdominal Hysterectomy This table compares the demographic and clinical characteristics of patients based on the dressing time after surgery, categorized into 12 hours and 48 hours groups. It presents the mean, standard deviation (SD), and median values for age, height, weight, BMI, and other variables. The p-value indicates the statistical significance of differences between the two dressing times. The symbols “(-)” and “(+)” represent the absence or presence of certain conditions, such as parity, abortion, curettage, smoking, additional disease, and indications for hysterectomy. The tests used for analysis include the independent samples t-test (t), Mann-Whitney U test (m), and chi-square test (X²). The results help in understanding if the dressing time has any significant impact on various postoperative outcomes.

Variable	Mean (12 Hours)	SD (12 Hours)	Median (12 Hours)	Mean (48 Hours)	SD (48 Hours)	Median (48 Hours)	Frequency (12 Hours)	% (12 Hours)	Frequency (48 Hours)	% (48 Hours)	p-value	Test
Age	47.4	5.4	48.0	48.2	6.5	48.0	-	-	-	-	0.692	m
Height	160.7	5.9	160.0	160.5	7.1	160.0	-	-	-	-	0.978	m
Weight	75.3	13.8	75.0	77.3	15.2	75.0	-	-	-	-	0.650	m
BMI	29.2	5.4	28.8	30.1	6.1	29.5	-	-	-	-	0.375	m
Parity (-)	-	-	-	-	-	-	11	11.0	8	8.0	0.469	X²
Parity (+)	-	-	-	-	-	-	89	89.0	92	92.0	0.469	X²
Number of Parities	2.7	1.4	2.0	2.6	1.4	2.0	-	-	-	-	0.297	m
Abortion (-)	-	-	-	-	-	-	83	83.0	73	73.0	0.088	X²
Abortion (+)	-	-	-	-	-	-	17	17.0	27	27.0	0.088	X²
Number of Abortions	1.6	1.3	1.0	1.7	1.3	1.0	-	-	-	-	0.718	m
Curettage (-)	-	-	-	-	-	-	94	94.0	90	90.0	0.297	X²
Curettage (+)	-	-	-	-	-	-	6	6.0	10	10.0	0.297	X²
Number of Curettages	1.7	0.8	1.5	1.2	0.6	1.0	-	-	-	-	0.116	m
Smoking (-)	-	-	-	-	-	-	91	91.0	96	96.0	0.152	X²
Smoking (+)	-	-	-	-	-	-	9	9.0	4	4.0	0.152	X²
Additional Disease (-)	-	-	-	-	-	-	69	69.0	56	56.0	0.058	X²
Additional Disease (+)	-	-	-	-	-	-	31	31.0	44	44.0	0.058	X²
Indications for Hysterectomy - Myoma Uteri	-	-	-	-	-	-	78	78.0	85	85.0	0.073	X²
Indications for Hysterectomy - Abnormal Uterine Bleeding	-	-	-	-	-	-	21	21.0	11	11.0	0.073	X²
Indications for Hysterectomy - Adnexal Mass	-	-	-	-	-	-	1	1.0	4	4.0	0.073	X²
Previous Operation (-)	-	-	-	-	-	-	39	39.0	41	41.0	0.773	X²
Previous Operation (+)	-	-	-	-	-	-	61	61.0	59	59.0	0.773	X²

Analyses related to parity rate and number, abortion rate and number, and curettage rate and number also found no statistically significant differences between the groups with dressing applications (p>0.05; Table 3). The evaluations of smoking rates and the presence of additional illnesses did not show significant differences between the groups (p>0.05; Table 3).

Comparisons of indications for hysterectomy and the presence of previous surgeries revealed no statistically significant differences between the 12- and 48-hour dressing groups (p>0.05; Table 3), indicating that these variables did not have a significant impact on dressing time in our study.

However, preoperative HGB values were found to be significantly lower in the group with 48-hour dressing applications compared to the group with 12-hour dressing applications (p<0.05; Table 4). No significant differences were detected in postoperative HGB values between the two groups (p>0.05; Table 4), and no significant differences were observed in preoperative and postoperative HCT values between the groups (p>0.05; Table 4).

**Table 4:** Outcomes of Dressing Removal Timing on Hemoglobin Levels, Hospital Stay, and Postoperative Complications This table provides a comparison of preoperative and postoperative hemoglobin and hematocrit levels, hospital stay duration, and the incidence of various post-surgical complications, categorized by two different dressing times: 12 hours and 48 hours. The mean, standard deviation (SD), median values, and percentages are given for each variable, along with the p-value to assess statistical significance. The “(-)” and “(+)” symbols indicate the absence or presence of complications such as hematoma, wound separation, superficial surgical site infection (SSI), and seroma. The table also includes readmission rates to the hospital. Statistical tests used are the independent samples t-test (T), Mann-Whitney U test (M), and chi-square test (X²), helping to determine if there are significant differences between the two groups.

Variable	12-Hour Dressing Mean	12-Hour Dressing SD	12-Hour Dressing Median	48-Hour Dressing Mean	48-Hour Dressing SD	48-Hour Dressing Median	p-value	Test Type
Preoperative Hemoglobin	11.9	1.5	12.1	11.4	1.9	11.4	0.029	T
Postoperative Hemoglobin	11.0	1.5	10.8	10.7	1.4	10.6	0.159	T
Preoperative Hematocrit	35.9	3.8	36.0	35.1	4.7	35.1	0.172	M
Postoperative Hematocrit	32.8	3.6	32.7	32.2	3.8	32.0	0.121	M
Hospital Stay Duration	52.1	10.1	-	54.7	10.3	54.8	0.002	M
Hematoma (-)	-	-	95%	-	-	97%	0.470	χ²
Hematoma (+)	-	-	5%	-	-	3%	0.470	χ²
Wound Separation (-)	-	-	91%	-	-	90%	0.809	χ²
Wound Separation (+)	-	-	9%	-	-	10%	0.809	χ²
Superficial SSI (-)	-	-	90%	-	-	90%	1.000	χ²
Superficial SSI (+)	-	-	10%	-	-	10%	1.000	χ²
Seroma (-)	-	-	90%	-	-	88%	0.651	χ²
Seroma (+)	-	-	10%	-	-	12%	0.651	χ²
Readmission to Hospital (-)	-	-	93%	-	-	92%	0.788	χ²
Readmission to Hospital (+)	-	-	7%	-	-	8%	0.788	χ²

Regarding the duration of hospital stay, the group with 48-hour dressing applications had significantly longer stays compared to the group with 12-hour dressing applications (p<0.05; Table 4). Analyses of hematoma, wound separation, superficial SSI, and seroma rates found no statistically significant differences between groups with dressing applications (p>0.05; Table 4). When comparing readmission rates, no significant differences were found between the groups with dressing applications (p>0.05; Table 4).

## Discussion

The success of surgical interventions is not limited to the technical proficiency of the operation; the quality of postoperative care is also vital in the recovery process. Surgical wound dressing, as a fundamental component of this process, plays a critical role in reducing the risk of SSIs. However, research on the optimal use and effects of dressing, due to methodological limitations and contradictory results, has prevented a clear consensus in this area. This situation is often limited by small sample sizes and high risks of bias, highlighting the importance of making dressing choices considering patient preferences and costs [9].

The complexity of the wound healing process and the multitude of dressing types available on the market have led to a lack of full understanding of wound care and management. This indicates the need for more high-quality research to evaluate the effectiveness of dressing [10,11].

This study aims to fill this gap by assessing the impact of dressing applications on SSI and exploring the potential benefits of integration with the ERAS protocol. Our ultimate goal is to understand the effects of dressing timing and the implementation of the ERAS protocol on the postoperative recovery process and to translate this information into actionable recommendations for clinical practice.

The study conducted by Toon et al. has shown that early removal of dressing from clean or clean-contaminated surgical wounds does not have a detrimental effect; it can even reduce hospital stay duration and treatment costs, although more research is needed in this area [12]. A meta-analysis examined whether early or late dressing removal, following primary closure of clean or clean contaminated surgical wounds, provided additional benefit to wound healing. This meta-analysis found that the timing of dressing removal, whether early (e.g., 24-48 hours after surgery) or late (e.g., several days later), did not create a significant difference in wound infection rates, wound healing time, and other clinical outcomes [13]. A large-scale study examining the timing of dressing removal after cesarean delivery observed that early (24 hours after surgery) or late (several days after surgery) dressing removal did not significantly affect parameters such as wound infection rates, healing time, and wound healing quality [14]. In light of these findings, current clinical studies support the conclusion that the duration of dressing wear does not have a decisive effect on SSI rates. This suggests that dressing applications and protocols require a more comprehensive approach to optimize wound healing and reduce the risk of SSI [15]. Consequently, the decision on how long to wear a dressing should be based on factors other than infection risk, such as patient comfort, dressing costs, and clinical protocols [16].

Our study has evaluated the impact of the duration of postoperative dressing application on postoperative complications such as hematoma formation, wound separation, superficial surgical site infection, and seroma. These complications are considered significant indicators of the postsurgical healing process, and the effect of the duration of dressing application on these complications could play an important role in clinical decision-making processes (Table 2).

A recent study has shown that dressing removal in the early postoperative period is safe and contributes to functional recovery in patients. However, this study also indicates that frequent dressing changes can negatively affect the ability of patients to move independently early on [17]. A multicenter, randomized controlled trial conducted by Kilic et al. [18] found that dressing removal 24 hours after low-risk cesarean deliveries, compared to waiting 48 hours, supported wound healing more effectively. These results can be interpreted as the duration of the 24-hour dressing that reduces microbial colonization in the wound area, limits inflammation, and accelerates the healing process. Furthermore, this approach has been determined to contribute to a more efficient use of hospital resources by allowing earlier discharge of patients [18]. The study by Vijayakumar et al. found that early removal of dressing significantly reduced the incidence of superficial SSI in clean and contaminated midline laparotomy wounds. It also significantly reduced the time required for complete wound healing and facilitated earlier discharge of patients compared to late dressing removal [19].

Our study, in accordance with the literature, has shown that there is no statistically significant difference between the groups with 12-hour and 48-hour dressing applications regarding the rates of hematoma, wound separation, superficial SSI, and seroma (p>0.05). These findings suggest that the duration of dressing application does not have a significant effect on these complications. In addition, these results could provide a basis for reviewing dressing protocols and possibly determining more flexible application durations. These findings can be considered an important step towards the development and improvement of postoperative care protocols (Table 4).

Early mobilization is supported by various protocols such as ERAS, and the implementation of these protocols improves the recovery process and helps patients return to normal more quickly [20]. When systematically integrated into gynecological surgical practices, the ERAS protocol is expected to contribute to shorter hospital stays and other positive clinical outcomes [21]. This protocol, designed to accelerate the postoperative recovery process, includes various evidence-based interventions covering preoperative, intraoperative, and postoperative periods. Managed by a multidisciplinary team, ERAS provides results that improve the recovery process, such as pain reduction, minimization of complication risks, and shorter hospital stays [22]. The application of the ERAS protocol in invasive procedures such as gynecological surgeries can positively affect the general health status of patients and optimize the rehabilitation process by reducing the physiological stress of surgery [23]. The study conducted by Mundhra et al. has shown that the ERAS protocol implemented after emergency cesarean sections significantly shortened hospital stays and improved patient quality of life. The ERAS protocol has been shown to allow faster healing and earlier discharge of patients, offering notable benefits, especially for patients living in challenging geographical conditions [24]. These findings suggest that the ERAS protocol could be an effective tool for improving post-cesarean care standards. The systematic review by Castelino et al. has revealed that the number of studies evaluating the impact of early mobilization protocols after abdominal surgery on postoperative outcomes is insufficient and the methodological quality of existing studies is low; moreover, the results obtained from these studies have been contradictory [25]. The research by Hu et al. emphasizes that promoting early mobilization in patients who have undergone abdominal surgery accelerates recovery through evidence-based practices, and the continuation of these practices is important [26]. The implementation of the ERAS protocol in patients undergoing abdominal hysterectomy can accelerate the recovery process while reducing hospital stays without increasing complication and readmission rates. This contributes significantly to the development of postoperative care protocols and the improvement of patient outcomes. Future studies should further illuminate the effects of the ERAS protocol on different surgical procedures and patient populations, revealing the potential for a broader application of this protocol [27].

Our study is one of the pioneering investigations on the effect of early dressing removal after abdominal hysterectomy on wound complications. The study has evaluated the impact of dressing application durations on postoperative complications and hospital stay durations. Consistent with similar studies in the literature and the fundamental principles of the ERAS protocol, our findings have observed significantly shorter hospital stays in the group with 12-hour dressing applications compared to the group with 48-hour applications (p<0.05). This finding underscores the potential of dressing application durations to improve the effectiveness of the ERAS protocol in the postoperative recovery process (Table 4).

Furthermore, our analyses have shown that there is no statistically significant difference between groups with dressing applications of 12 and 48 hours in terms of hospital readmission rates (p>0.05). This finding suggests that early dressing removal does not have a negative impact on wound healing and supports patients’ ability to move independently early on. These results indicate that postoperative care protocols and dressing application processes must be reviewed, and dressing application durations aligned with the ERAS protocol could effectively reduce hospital stays (Table 4).

This study demonstrates that optimizing the duration of dressing application after abdominal hysterectomy can contribute to the patient’s recovery process and allow more efficient use of the resources of the healthcare system. Determining the duration of dressing application in accordance with the ERAS protocol can accelerate the healing process and reduce the risk of postoperative complications. Therefore, future studies are expected to examine the effects of dressing application durations that comply with the ERAS protocol on different surgical procedures and patient populations in more detail, expanding the scope of the application of this protocol.

Postoperative abdominal hysterectomy SSIs are associated with factors such as increased BMI, blood transfusions, and the duration of the operation. These factors can marginally increase the risk of infection, necessitating surgeons to have in-depth knowledge of anatomy, surgical risks, prophylaxis, and prevention methods [28]. The implementation of the ERAS protocol has positive effects on the quality of recovery in patients after planned hysterectomy. This protocol is considered an essential component of modern surgical practices and aims to raise postoperative care standards, increase patient satisfaction, and improve the efficiency of health systems [29]. The ERAS protocol is a multidisciplinary approach designed to accelerate the postoperative recovery process and improve the overall quality of patient recovery.

However, the lack of clear recommendations within the ERAS protocol regarding postoperative wound care, particularly when the dressing is opened can cause uncertainty in clinical practice [30]. This study has examined the impact of dressing timing after abdominal hysterectomy on patient demographic characteristics and clinical outcomes. The findings have shown that dressing timing did not have a significant effect on most parameters, indicating that early or late dressing removal timing did not make a significant difference in expected clinical outcomes. These results suggest that the dressing time within the ERAS protocol could be more flexible, and other factors should also be considered in the clinical decision-making process. Future studies could lay the groundwork for adding postoperative wound care guidelines to the ERAS protocol and evaluating the effectiveness of these guidelines in clinical practice.

Our study has demonstrated that the timing of dressing removal does not have a significant effect on demographic and clinical characteristics such as age, height, weight, and BMI, as well as childbirth and abortion history, smoking habits, and the presence of additional diseases. The indications for hysterectomy and the history of previous surgeries, among other factors, do not need to be considered when deciding on the timing of dressing removal (Tables 1-3).

Upon a more detailed examination of this study, it emerges as one of the pioneering investigations on the effect of early dressing removal on wound complications after abdominal hysterectomy. The study has evaluated the impact of dressing application durations on postoperative complications and hospital stay lengths, finding that, consistent with similar studies in the literature and the fundamental principles of the ERAS protocol, groups with 12-hour dressing applications experienced significantly shorter hospital stays compared to those with 48-hour applications (p<0.05). Furthermore, the analyses conducted within the study have revealed no statistically significant differences between the 12- and 48-hour dressing application groups in terms of hospital readmission rates (p>0.05). These findings suggest that the timing of dressing application does not have a significant impact on postoperative complications and that, within the ERAS protocol, the timing of dressing application could be more flexible. These results imply that determining the duration of dressing application in accordance with the ERAS protocol could accelerate the healing process for patients and reduce the risk of postoperative complications (Table 4).

The potential limitations of this study include the reliance on the software algorithm for randomization and the specific criteria set for participant selection. These factors could impact the generalizability of the results and raise questions regarding their extrapolation to other populations or conditions. Moreover, small sample sizes and high risks of bias have contributed to the ongoing debate over the optimal use and effects of dressings in research. The wide array of dressing types available has hindered a comprehensive understanding of wound care and management, underscoring the necessity for more rigorous research to evaluate dressing effectiveness. This study aims to augment the current literature in this domain, offering essential insights to enhance wound care practices.

## Conclusions

These findings indicate that the timing of dressing removal does not have a significant effect on the parameters examined. Therefore, when deciding when to remove dressings, factors such as patient comfort, cost, and clinical protocols should also be considered alongside these parameters. Furthermore, the addition of postoperative wound care guidelines to the ERAS protocol and the evaluation of the effectiveness of these guidelines in clinical practice have been identified as important areas for future research. More evidence-based research on dressing selection and timing is critical to optimizing wound healing and reducing the risk of infection.

## References

[1] García-Gubern CF, Colon-Rolon L, Bond MC (2010). Essential concepts of wound management. Emerg Med Clin North Am.

[2] Wallace HA, Basehore BM, Zito PM (2023). Stages of wound healing. StatPearls.

[3] Peleg D, Eberstark E, Warsof SL, Cohen N, Ben Shachar I (2016). Early wound dressing removal after scheduled cesarean delivery: a randomized controlled trial. Am J Obstet Gynecol.

[4] Mendes DA, Veiga DF, Veiga-Filho J (2018). Influence of dressing application time after breast augmentation on cutaneous colonization: a randomized clinical trial. J Plast Reconstr Aesthet Surg.

[5] Zuarez-Easton S, Zafran N, Garmi G, Salim R (2017). Postcesarean wound infection: prevalence, impact, prevention, and management challenges. Int J Womens Health.

[6] Manandhar T, Sitaula S, Thapa BD, Agrawal A, Thakur A (2020). Prevalence of hysterectomy among gynecological surgeries in a tertiary care hospital. JNMA J Nepal Med Assoc.

[7] Khapre SS, Joshi V, Hivre MD (2024). Hysterectomy profile in King Edward Memorial Hospital, Pune, India: indications, routes of surgery, and complications. Cureus.

[8] Piovano E, Pagano E, Del Piano E (2022). Implementation of the ERAS (Enhanced Recovery After Surgery) protocol for hysterectomy in the Piedmont Region with an audit&feedback approach: Study protocol for a stepped wedge cluster randomized controlled trial. A study of the EASY-NET project. PLoS One.

[9] Dumville JC, Gray TA, Walter CJ (2016). Dressings for the prevention of surgical site infection. Cochrane Database Syst Rev.

[10] Sood A, Granick MS, Tomaselli NL (2014). Wound dressings and comparative effectiveness data. Adv Wound Care (New Rochelle).

[11] Blackburn J, Ousey K, Stephenson J (2019). Nurses' education, confidence, and competence in appropriate dressing choice. Adv Skin Wound Care.

[12] Toon CD, Lusuku C, Ramamoorthy R, Davidson BR, Gurusamy KS (2015). Early versus delayed dressing removal after primary closure of clean and clean-contaminated surgical wounds. Cochrane Database Syst Rev.

[13] Zhang T, Zhang F, Chen Z, Cheng X (2020). Comparison of early and delayed removal of dressing following primary closure of clean and contaminated surgical wounds: a systematic review and meta-analysis of randomized controlled trials. Exp Ther Med.

[14] Heinemann N, Solnica A, Abdelkader R, Gutman J, Nalbandian N, Raizman E, Hochner-Celnikier D (2019). Timing of staples and dressing removal after cesarean delivery (the SCARR study). Int J Gynaecol Obstet.

[15] Veiga DF, Damasceno CA, Veiga-Filho J (2016). Dressing wear time after breast reconstruction: a randomized clinical trial. PLoS One.

[16] Ammar G, Almashaikh E, Ibdah A (2019). Impact of early dressing removal on tunneled central venous catheters: a piloting study. Asian Pac J Cancer Prev.

[17] Atlan F, Ashkenazi I, Shehadeh K (2021). Early postoperative dressing removal in hand surgery: novel concepts for individualized surgical dressing management. Hand Surg Rehabil.

[18] Kilic GS, Demirdag E, Findik MF (2021). Impact of timing on wound dressing removal after caesarean delivery: a multicentre, randomised controlled trial. J Obstet Gynaecol.

[19] Vijayakumar C, Prabhu R, Muthukrishnan V, Kalaiarasi R, Swetha T (2018). Early versus late dressing removal in clean and contaminated midline laparotomy wounds: a non-randomized pilot study. Int Surg J.

[20] Borges MG, Borges DL, Ribeiro MO, Lima LS, Macedo KC, Nina VJ (2022). Early mobilization prescription in patients undergoing cardiac surgery: systematic review. Braz J Cardiovasc Surg.

[21] Wijk L, Franzen K, Ljungqvist O, Nilsson K (2014). Implementing a structured Enhanced Recovery After Surgery (ERAS) protocol reduces length of stay after abdominal hysterectomy. Acta Obstet Gynecol Scand.

[22] Willner A, Teske C, Hackert T, Welsch T (2023). Effects of early postoperative mobilization following gastrointestinal surgery: systematic review and meta-analysis. BJS Open.

[23] Scheib SA, Thomassee M, Kenner JL (2019). Enhanced recovery after surgery in gynecology: a review of the literature. J Minim Invasive Gynecol.

[24] Mundhra R, Gupta DK, Bahadur A, Kumar A, Kumar R (2024). Effect of Enhanced Recovery after Surgery (ERAS) protocol on maternal outcomes following emergency caesarean delivery: a randomized controlled trial. Eur J Obstet Gynecol Reprod Biol X.

[25] Hu Y, McArthur A, Yu Z (2019). Early postoperative mobilization in patients undergoing abdominal surgery: a best practice implementation project. JBI Database System Rev Implement Rep.

[26] Castelino T, Fiore JF Jr, Niculiseanu P, Landry T, Augustin B, Feldman LS (2016). The effect of early mobilization protocols on postoperative outcomes following abdominal and thoracic surgery: a systematic review. Surgery.

[27] Kalogera E, Dowdy SC (2016). Enhanced recovery pathway in gynecologic surgery: improving outcomes through evidence-based medicine. Obstet Gynecol Clin North Am.

[28] Olsen MA, Higham-Kessler J, Yokoe DS (2009). Developing a risk stratification model for surgical site infection after abdominal hysterectomy. Infect Control Hosp Epidemiol.

[29] Hodges KR, Davis BR, Swaim LS (2014). Prevention and management of hysterectomy complications. Clin Obstet Gynecol.

[30] Martin F, Vautrin N, Elnar AA, Goetz C, Bécret A (2022). Evaluation of the impact of an enhanced recovery after surgery (ERAS) programme on the quality of recovery in patients undergoing a scheduled hysterectomy: a prospective single-centre before-after study protocol (RAACHYS study). BMJ Open.

